# Vagal Neuromodulation in Chronic Heart Failure With Reduced Ejection Fraction: A Systematic Review and Meta-Analysis

**DOI:** 10.3389/fcvm.2021.766676

**Published:** 2021-11-24

**Authors:** Lucas Bonacossa Sant'Anna, Sérgio Lívio Menezes Couceiro, Eduardo Amar Ferreira, Mariana Bonacossa Sant'Anna, Pedro Rey Cardoso, Evandro Tinoco Mesquita, Guilherme Mendes Sant'Anna, Fernando Mendes Sant'Anna

**Affiliations:** ^1^Medical School, Department of Education and Graduation, Fundação Técnico-Educacional Souza Marques, Rio de Janeiro, Brazil; ^2^Hospital Santa Izabel, Rio de Janeiro, Brazil; ^3^Department of Cardiology, Universidade Federal Fluminense, Niterói, Brazil; ^4^Neonatal Division, McGill University Health Center, Montreal, QC, Canada; ^5^Department of Education and Graduation, Universidade Federal do Rio de Janeiro, Rio de Janeiro, Brazil

**Keywords:** chronic heart failure, vagal nerve stimulation, reduced ejection fraction, NYHA class, 6 min walk distance (6 MWD)

## Abstract

**Objectives:** The aim of this study was to evaluate the effects of invasive vagal nerve stimulation (VNS) in patients with chronic heart failure (HF) and reduced ejection fraction (HFrEF).

**Background:** Heart failure is characterized by autonomic nervous system imbalance and electrical events that can lead to sudden death. The effects of parasympathetic (vagal) stimulation in patients with HF are not well-established.

**Methods:** From May 1994 to July 2020, a systematic review was performed using PubMed, Embase, and Cochrane Library for clinical trials, comparing VNS with medical therapy for the management of chronic HFrEF (EF ≤ 40%). A meta-analysis of several outcomes and adverse effects was completed, and GRADE was used to assess the level of evidence.

**Results:** Four randomized controlled trials (RCT) and three prospective studies, totalizing 1,263 patients were identified; 756 treated with VNS and 507 with medical therapy. RCT data were included in the meta-analysis (fixed-effect distribution). Adverse effects related to VNS were observed in only 11% of patients. VNS was associated with significant improvement (GRADE = High) in the New York Heart Association (NYHA) functional class (OR, 2.72, 95% CI: 2.07–3.57, *p* < 0.0001), quality of life (MD −14.18, 95% CI: −18.09 to −10.28, *p* < 0.0001), a 6-min walk test (MD, 55.46, 95% CI: 39.11–71.81, *p* < 0.0001) and NT-proBNP levels (MD −144.25, 95% CI: −238.31 to −50.18, *p* = 0.003). There was no difference in mortality (OR, 1.24; 95% CI: 0.82–1.89, *p* = 0.43).

**Conclusions:** A high grade of evidence demonstrated that vagal nerve stimulation improves NYHA functional class, a 6-min walk test, quality of life, and NT-proBNP levels in patients with chronic HFrEF, with no differences in mortality.

**Graphical Abstract:**
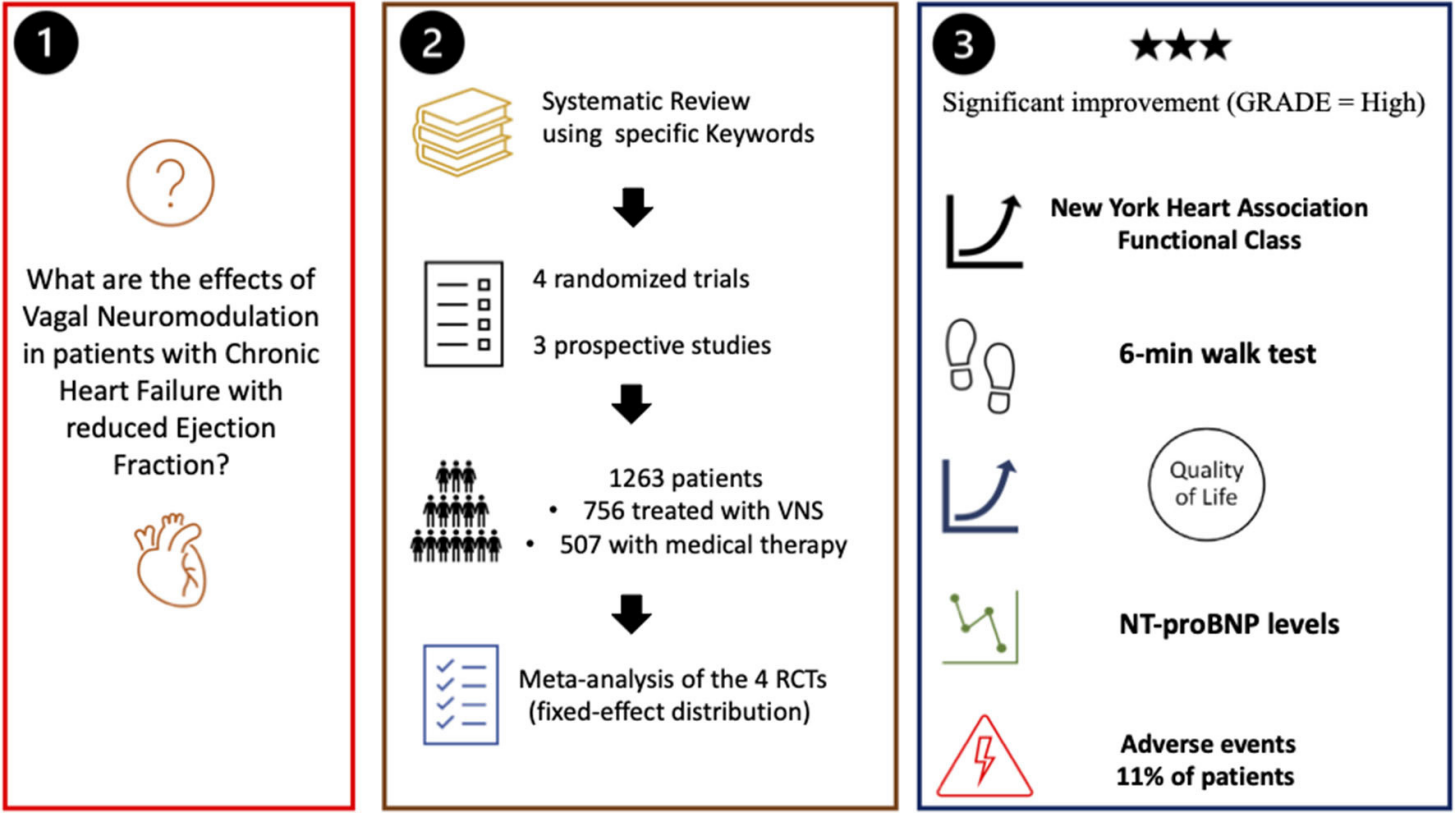
Summary of the study.

## Introduction

The autonomic nervous system (ANS) is responsible for the homeostatic balance of the human body, notably on the cardiac and gastrointestinal systems. ANS has two main components: the sympathetic and parasympathetic systems. In the heart, parasympathetic system activation decreases the frequency, contractility, conductance, and O_2_ consumption, leading to a drop in cardiac output with relaxation and rest of the heart (1). The vagus nerve (10th cranial pair) is responsible for most of the parasympathetic innervation, including all major thoracic organs (2).

Autonomic nervous system imbalances have been observed in a diverse range of diseases and health problems and, in most cases, are associated with increased sympathetic and decreased parasympathetic tone (3), such as in heart failure (4), inflammatory bowel diseases (5), and chronic pain syndrome (6). Thus, the idea of using vagal stimulation to increase parasympathetic activity to treat some of these diseases was first introduced by James Corning in the late nineteenth century (7). Currently, vagal stimulation is approved by the US Food and Drug Administration (FDA) for treatment of epilepsy (8) and treatment-resistant depression (8), and recently, it has been also approved to treat episodic cluster headaches (9).

The imbalance of the ANS and the heart failure (HF) creates a vicious cycle; the excess of sympathetic activity and the withdrawal of vagal activity clearly contribute to the progression of ventricular remodeling and worsening of heart failure, and *vice versa*, the progression of HF could augment the imbalance between sympathetic and vagal activity (10). The enhanced sympathetic activity can be regulated by drugs of beta-adrenergic blockade or inhibitors of the renin–angiotensin–aldosterone system, and reduced parasympathetic activity can be maintained by physical training, for example. However, the pace of new drug therapies has declined significantly. Several relatively new and experimental non-pharmacological interventions, which target specific aspects of autonomic imbalance (cervical vagus nerve stimulation, renal denervation, spinal cord stimulation, and carotid sinus nerve stimulation), are being actively investigated nowadays. All in all, autonomic neuromodulation was the key target in HF treatment, and device therapy to achieve autonomic modulation has garnered significant interest.

Although heart failure is associated with ANS imbalance, the beneficial effects of deep or transcutaneous vagal stimulation in these patients remain unclear despite some randomized controlled trials (RCT) that have been conducted (11). Moreover, all studies using invasive vagal nerve stimulation (VNS) for heart failure involved patients with reduced ejection fraction, which is the most common and serious presentation of HF. Therefore, the aim of the present study was to perform a systematic review and meta-analysis on the effects of invasive VNS in patients with chronic heart failure and reduced ejection fraction (HFrEF).

## Methods

This systematic review and meta-analysis being reported according to Preferred Reported Items for Systematic Reviews and Meta-Analysis (PRISMA) (12) guidelines and remain in accordance with specific regulations for non-randomized studies. A protocol for this systematic review was developed *a priori* and registered in PROSPERO under the number CRD42021232377.

### Data Sources and Search Strategies

Initially, a search was conducted for similar meta-analyses on the cardiac effects of vagal stimulation for the treatment of heart failure. This initial search was carried out on MEDLINE (PubMed) and Embase; one meta-analysis published only as an abstract was found (13). The same search was then performed in the aforementioned platforms and the Cochrane Library, using the MeSH terms (“Vagus Nerve Stimulation” OR “Vagal Nerve Stimulation” OR “VNS” OR “Baroreflex Activation”) AND (“Heart Failure” OR “Cardiac Failure” OR “CHF” OR “Chronic Heart Failure” OR “Congestive Heart Failure”), to search for clinical trials (randomized or not) conducted in humans between May 1994 and July 2020 (Table 1).

**Table 1:** Search strategy.

**Source**	**MeSH terms**	**Date**	**Results**
MEDLINE	(Vagus Nerve Stimulation OR Vagal Nerve Stimulation OR VNS OR Baroreflex Activation) AND (Heart Failure OR Cardiac Failure OR CHF OR Chronic Heart Failure OR Congestive Heart Failure)	From May 1994 to July 11th, 2020	762
EMBASE	(Vagus Nerve Stimulation OR Vagal Nerve Stimulation OR VNS OR Baroreflex Activation) AND (Heart Failure OR Cardiac Failure OR CHF OR Chronic Heart Failure OR Congestive Heart Failure)	From May 1994 to July 11th, 2020	1494
Cochrane library of trials	(Vagus Nerve Stimulation OR Vagal Nerve Stimulation OR VNS OR Baroreflex Activation) AND (Heart Failure OR Cardiac Failure OR CHF OR Chronic Heart Failure OR Congestive Heart Failure)	From May 1994 to July 11th, 2020	122

### Study Selection

Studies were selected according to any of the following criteria: (1) measurements of the effects of vagal stimulation in patients with chronic heart failure and reduced left ventricular ejection fraction (LVEF ≤ 40%) on (a) heart rate; (b) ejection fraction (reflecting left ventricular function); (c) left ventricular end-systolic and end-diastolic volume (LVESV and LVEDV); (d) six-minute walking test (6-min WT); (e) quality of life (QoL); (f) changes in NYHA (New York Heart Association) functional class; and (g) NT pro-B-type natriuretic peptide (NT-proBNP) levels; (2) clear description ofVNS; (3) other interventions, if present, well-discriminated and described; (4) monitoring of results; and (5) clear description of adverse effects.

The key exclusion criteria of the studies were: persistent or permanent atrial fibrillation, cardiac resynchronization (CRT) for <1 year or a QRS of >130 ms without CRT, type I diabetes, type II diabetes for >5 years, sleep disordered breathing that had been treated for <6 months, a surgically correctable cause of HF, recent HF hospitalization or myocardial infarction (30 or 90 days, respectively), or an indication for dialysis, cardiac surgery in the preceding 6 months, and severe liver or renal failure. One trial (14) recruited patients with CRT but persistent NYHA functional class III, and another one also included patients with atrial fibrillation (15).

In most studies, effectiveness endpoints were the change from the baseline to 6 months in 6-min walk distance, Minnesota Living with HF Questionnaire quality-of-life (QOL) score, NYHA functional class, and N-terminal pro-B-type natriuretic peptide (NT-proBNP) levels. Only one study (14) evaluated death from any cause or first event for worsening HF.

The initial list of articles generated was initially filtered for clinical trials, and then duplicates were removed. The selected titles had the abstracts analyzed. All these steps were done by three researchers (LBS, FMS, and EAF) using the above-described criteria. Disagreements between investigators were solved by a discussion with a senior researcher (FMS). Finally, all studies selected were read by all the authors to confirm eligibility, and results were tabulated according to the specific description of the selected studies and reviewed for further statistical analysis.

### Assessment of Study Quality

Individual study quality was assessed by three reviewers (SLMC, FMS, and LBS) using the revised Cochrane risk-of-bias tool for randomized trials (RoB 2.0) across five domains (randomization, intended intervention, missing data, outcome, measurement, and reported results) (16) (Table 2). The quality of evidence was rated by the Grading of Recommendation, Assessment, Development and Evaluation (GRADE) process (17). Confidence in the estimate of the primary outcome was based on five domains, the risk of bias, inconsistency, indirectness, imprecision, and other considerations, and was categorized into four levels, from very low (⊕⊖⊖⊖) to high (⊕⊕⊕⊕) (Table 3). Any differences were resolved by discussion until consensus was reached.

**Table 2:** Risk of bias of the included randomized controlled trials.

	**Zannad**	**Abraham**	**Gold**	**Zile**
Adequate sequence generation?	+	+	+	+
Allocation concealment?	+	+	+	+
Blinding of participants and personnel (performance bias)?	+	–	–	–
Blinding of outcome assessment (detection bias)?	+	+	+	+
Incomplete outcome data assessed?	+	+	–	+
Free of selective reporting?	+	+	+	+

*+, Yes; −, No*.

**Table 3:** Summary of findings.

**Vagal nerve stimulation plus usual care compared to usual care for heart failure with reduced ejection fraction**						
**Patient or population:** heart failure with reduced ejection fraction						
**Setting:** chronically stable patients enrolled in multiple centers in USA, Europe and Canada						
**Intervention:** vagal nerve stimulation plus usual care						
**Comparison:** usual care						
**Outcomes**	Anticipated absolute effects^*^ **(95% CI)**		**Relative effect (95% CI)**	**No of participants (studies)**	**Certainty of the evidence (GRADE)**	**Comments**
	**Risk with usual care**	**Risk with vagal nerve stimulation plus usual care**				
MortalityFollow up: median 6 months	81 per 1,000	**96 per 1,000** (66–138)	**OR 1.2** (0.80–1.82)	1,206 (4 RCTs)	⊕⊕⊕⊕ HIGH	VNS has no effect on mortality.
NYHA functional classFollow up: median 6 months	304 per 1,000	**543 per 1,000** (474–609)	**OR 2.72** (2.07–3.57)	969 (4 RCTs)	⊕⊕⊕⊕ HIGH	There was an improvement of at least one NYHA functional class in VNS group.
Quality of lifeFollow up: median 6 months	The mean quality of life was **44.3**	MD **14.18 lower** (18.09 lower to 10.28 lower)	-	450 (3 RCTs)	⊕⊕⊕⊕ HIGH	Quality of life, assessed by the MLwHFQ (lesser is better), showed a consistent improvement in all RCTs.
6-min WTFollow up: median 6 months	The mean 6-min WT was **303.6** meters	MD **55.46 meters higher** (39.11 higher to 71.81 higher)	-	728 (3 RCTs)	⊕⊕⊕⊕ HIGH	6-min walking test distance significantly increased in all trials in VNS groups.
NT-proBNP (pg/ml)Follow up: median 6 months	The median NT-proBNP (pg/ml) was **970.5** pg/ml	MD **144.25 pg/ml lower** (238.31 lower to 50.18 lower)	-	445 (3 RCTs)	⊕⊕⊕⊕ HIGH	NP-proBNP levels (a biomarker of heart failure) decreased in most trials analyzed.

* *The risk in the intervention group (and its 95% confidence interval) is based on the assumed risk in the comparison group and the relative effect of the intervention (and its 95% CI)*.

*CI, Confidence interval; OR, Odds ratio; MD, Mean difference; NYHA, New York Heart Association; 6-min WT, 6-min walking test; MLwHFQ, Minnesota Living with Heart Failure Questionnaire; NT-proBNP, N-terminal-pro-brain natriuretic peptide*.

*GRADE Working Group grades of evidence*.

*High certainty: We are very confident that the true effect lies close to that of the estimate of the effect*.

*Moderate certainty: We are moderately confident in the effect estimate: The true effect is likely to be close to the estimate of the effect, but there is a possibility that it is substantially different*.

*Low certainty: Our confidence in the effect estimate is limited: The true effect may be substantially different from the estimate of the effect*.

*Very low certainty: We have very little confidence in the effect estimate: The true effect is likely to be substantially different from the estimate of effect*.

### Statistical Analysis

This meta-analysis was carried out with the software Review Manager (RevMan), version 5.4 (The Cochrane Collaboration, 2020). A funnel plot was used to assess for publication bias. A fixed effects model with inverse variance weighting was used to account for heterogeneity across studies, which was measured using the Cochrane *I*^2^ statistic: <25–50% = mild, 50–75% = moderate, and >75% = severe heterogeneity. Adjusted odds ratios (ORs) and mean differences (MD) with 95% CIs were pooled to evaluate prognosis.

Data were separated into two distinct groups for comparisons: VNS and no VNS (control) and compared by fixed-effect distribution tests. The variability of the results between the studies (τ^2^) was considered the same for the two groups, and differences between the groups were considered statistically significant if *p* < 0.05; all tests were two-tailed.

## Results

### Study Characteristics and Quality Assessment

**Figure 1:**
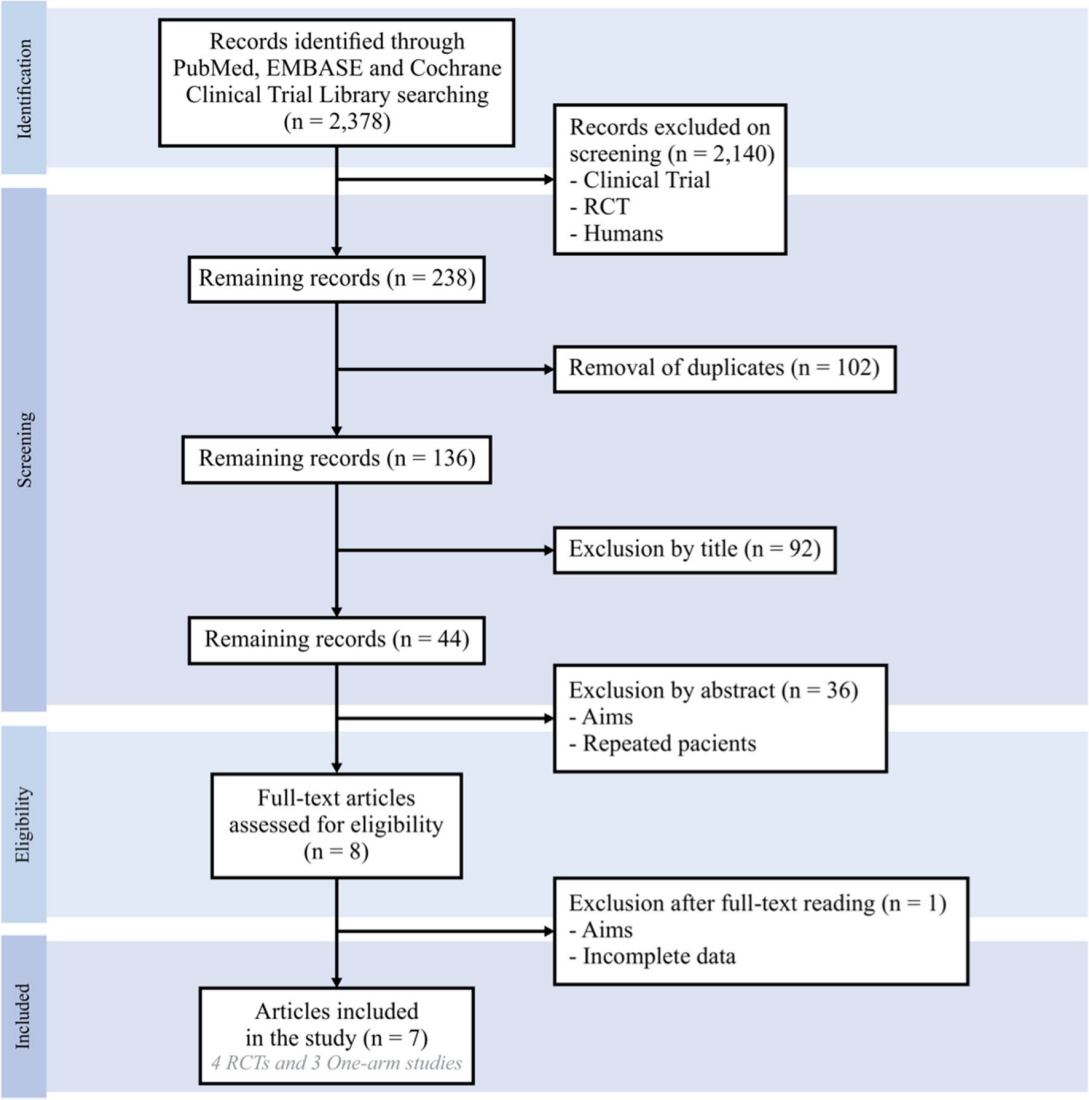
A study flowchart.

The initial search of articles by the MeSH terms used on the EMBASE, MEDLINE (PubMed), and Cochrane Library platforms returned 2,378 articles, which were filtered as “controlled clinical trial” OR “randomized clinical trial,” with 238 articles remaining. A total of 102 duplicates were removed, and the selection by titles excluded another 92 articles. Of the 44 remaining papers, 36 were removed after reading the abstracts. During the complete analysis of the manuscripts, another study was eliminated. Of the seven articles selected, three were prospective studies (4, 18, 19) and four were RCT (14, 15, 20, 21). Only data from the RCTs were included in the meta-analysis (Figure 1). The Egger's test and funnel plot showed no evidence of publication bias.

The four RCTs enrolled a total of 1,107 patients, of which 641 were treated with VNS: mean age was 62.1 years, 82.5% were male, and all patients were NYHA class ≥ II. The median follow-up was 6 months (range: 6–16 months). The methodology used was similar between the studies. There were no significant differences in the populations included and baseline characteristics of VNS and control groups (Tables 4, 5).

**Table 4:** Initial data of the randomized controlled trials.

	**Zannad et al**. **(****21****)**		**Abraham et al**. **(****20****)**		**Gold et al**. **(****14****)**		**Zile et al**. **(****15****)**	
	**VNS**	**Control**	**BAT**	**Control**	**VNS**	**Control**	**BAT**	**Control**
Initial size	63	32	76	70	436	271	130	134
Finally analyzed	59	28	71	69	391	244	120	125
Men (%)	89	81	87.3	84.1	77.8	80.8	82	78
Age (mean ± SD)	59.8 ± 12.2	59.3 ± 10.1	64 ± 11	66 ± 12	61.7 ± 10.5	60.9 ± 11.2	62 ± 11	63 ± 10
Main outcomes	LVESD		NYHA class, 6-min WT, QoL		Death or worsening of HF		NT-proBNP, 6-min WT, QoL	
Other outcomes	LVESV, LVEF, peak VO_2_, NT-proBNP		NT-proBNP, echo parameters		NYHA class, 6-min WT, QoL		NYHA class, death or HF hospitalization	
Follow-up	6 months		6 months		16 months		6 months	
NYHA II/III, *n*	7/52	7/21	1/70	0/69	0/436	0/271	9/121	7/127
QoL	44.4 ± 22.2	42.4 ± 25.1	51 ± 21	43 ± 22	51.6 ± 20.7	52.2 ± 21.8	53 ± 24	52 ± 24
6-min WT (m)	-	-	297 ± 79	308 ± 85	304 ± 111	317 ± 178	316 ± 68	294 ± 73
Heart rate	68.2 ± 13.2	71.3 ± 12.9	73 ± 11	75 ± 12	72.5 ± 12.2	71.4 ± 11.5	75 ± 10	75 ± 11
LVEF (%)	30.5 ± 6.0	30.8 ± 4.2	24 ± 7	25 ± 7	23.9 ± 6.7	25.2 ± 7.3	27 ± 7	28 ± 6
LVESD/LVEDD (cm)	4.9/5.9	5.2/6.0	-	-	-	-	-	-
NT-proBNP (pg/ml)	870 (370–1,843)	882 (488–1,926)	1,422 (455–4,599)	1,172 (548–2,558)	-	-	731 (475–1,021)	765 (479–1,052)
Mortality, *n* (%)	1 (1.6)	2 (6.3)	5 (6.6)	5 (7.1)	62 (14.2)	28 (10.3)	2 (1.5)	3 (2.2)
AERP, *n* (%)	9 (14.3)	4 (12.5)	10 (14.1)	-	37 (9.4)	-	4 (3.2)	-

*VNS, vagus nerve stimulation; BAT, baroreflex activation therapy; NYHA, New York Heart Association functional class; QoL, quality of life; 6-min WT: 6 min walking test (meters); LVEF, left ventricular ejection fraction; LVESD, left ventricular end systolic diameter; LVEDD, left ventricular end diastolic diameter; NT-proBNP, N-terminal pro-B-type natriuretic peptide; HF, heart failure; AERP, adverse effects related to the procedure*.

**Table 5:** Prospective studies before and after vagus nerve stimulation.

	**Schwartz et al**. **(****18****)** **(*****n*** **=** **8)**		**De Ferrari et al**. **(****4****)** **(*****n*** **=** **32)**		**Gronda** **(****19****)** **(*****n*** **=** **11)**	
	**Baseline**	**6-months**	**Baseline**	**6-months**	**Baseline**	**6-months**
Men	100%		94%		82.7%	
Age	54		56 ± 11		67 ± 9	
Main outcomes	All AERP		All AERP		MSNA, QOL, functional capacity	
Other outcomes	NYHA class, QoL, 6-min WT,		NYHA class, QoL, 6-min WT,		BNP, LVEF	
	LVEF, LVESV, LVEDV		LVEF, LVESV, LVEDV			
NYHA I/II/III/IV, n	0/1/7/0	1/3/4/0^*^	0/15/15/2	10/14/5/0^**^	0/0/11/0	8/2/1/0^**^
QoL	52 ± 14	31 ± 18^‡^	49 ± 17	32 ± 19^**^	33.4 ± 29.8	−10.6 ± 3.8^*^
6-min WT (m)	405 ± 43	446 ± 96^*^	411 ± 76	471 ± 111^**^	304.4 ± 49.6	+51.1 ± 25.6^*^
Heart rate	87 ± 13	83 ± 12^*^	82 ± 13	76 ± 13^‡^	72.3 ± 8.3	−0.5 ± 1.8
LVEF (%)	24 ± 5	26 ± 10	22.3 ± 6.9	28.7 ± 6.4^**^	32.0 ± 7.3	+3.6 ± 1.4^‡^
LVESV (ml)	208 ± 71	198 ± 83^*^	103 ± 35 ml/m^2^	89 ± 38 ml/m^2^^*^	116.9 ± 40.9	−11.3 ± 5.6^*^
LVEDV (ml)	273 ± 81	250 ± 82	132 ± 42 ml/m^2^	125 ± 46 ml/m^2^	168.6 ± 43.5	−8.7 ± 7.5
BNP, pg/ml	-	-	-	-	314.4 ± 306.9	+33.1 ± 112.3
MSNA (bursts/min)	-	-	-	-	45.1 ± 7.7	−13.8 ± 5.4^**^
Mortality, *n* (%)	0		3 (9.4)		0	
AERP, *n* (%)	1 (12.5)		5 (15.6)		1 (9.1)	

*NYHA, New York Heart Association functional class; QoL, quality of life; 6-min WT: 6 min walking test (meters); LVEF, left ventricular ejection fraction; LVESV, left ventricular end systolic volume; LVEDV, left ventricular end diastolic volume; MSNA, muscle sympathetic nerve activity; BNP, brain natriuretic peptide; HF, heart failure; AERP, adverse effects related to the procedure. Continuous data is presented as mean ± SD, except for Gronda, which is presented as mean ± SE*.

* *p < 0.05*.

‡ *p < 0.005*.

** *p < 0.001*.

### Outcomes

#### Mortality

**Figure 2:**
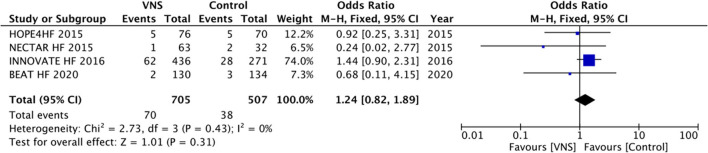
All-cause mortality during follow-up between vagal nerve stimulation and control groups for management of chronic heart failure with reduced ejection fraction. VNS, vagus nerve stimulation.

No differences in mortality between VNS and control groups (OR, 1.24; 95% CI, 0.82–1.89, *p* = 0.43) were detected (Figure 2).

#### Left Ventricular End-Systolic and Diastolic Volumes, Heart Rate, and Left Ventricular Ejection Fraction

LVESV was evaluated in only two of the RCTs and by using different methodologies. There were no differences between groups. In contrast, two prospective studies have shown a decrease in LVESV after VNS (18, 19) when compared to baseline measurements. No study depicted a statistically significant difference in LVEDV after treatment. Only two prospective studies evaluated HR after VNS and found a significant HR decrease [FIM, HR = 87 ± 13 (before) vs. 83 ± 12 (after 6 months), *p* = 0.01, and CARDIOFIT, HR = 85 ± 14 (before) vs. 76 ± 11 (after 1 year), *p* = 0.003]. All trials evaluated LVEF at the baseline, but only the prospective studies and 1 RCT (21) assessed it after 6 months. There was a significant improvement in LVEF in two prospective studies (4, 19), but not in the others.

#### NYHA Criteria

**Figure 3:**
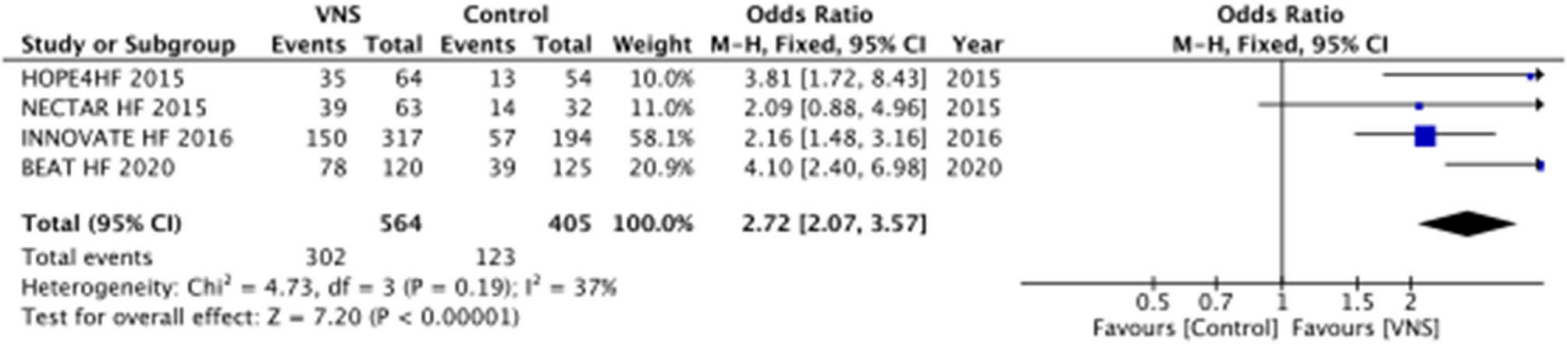
Improvement of at least one New York Heart Association functional class during follow-up between VNS and control groups for management of chronic heart failure with reduced ejection fraction. VNS, vagus nerve stimulation.

All trials evaluated NYHA criteria. A significant improvement of at least one point in the NYHA class could be observed in patients undergoing VNS for all studies analyzed. In the meta-analysis (Figure 3), the VNS group showed better class scores when compared to controls (OR, 2.72; 95% CI, 2.07–3.57; *p* < 0.0001).

#### Quality of Life

**Figure 4:**
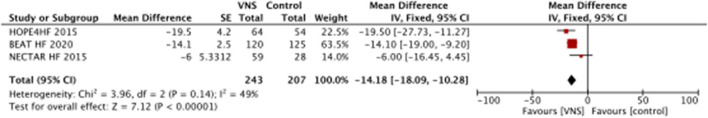
Improvement of quality of life during follow-up between VNS and control groups for management of chronic heart failure with reduced ejection fraction. VNS, vagus nerve stimulation.

Comparison between the quality of life (QoL) before and after treatment showed an improvement in its scores in all four randomized clinical trials and prospective studies (Tables 4, 5). Three of the randomized studies used the Minnesota Living with Heart Failure Questionnaire (MLwHFQ) (15, 20, 21), and one used the Kansas City Cardiomyopathy Questionnaire (KCCQ) (14). There was a significant improvement in QoL favoring VNS in all randomized studies (MD −14.18; 95% CI, −18.09 to −10.28; *p* < 0.0001) (Figure 4).

#### 6-min Walking Test Distance

**Figure 5:**
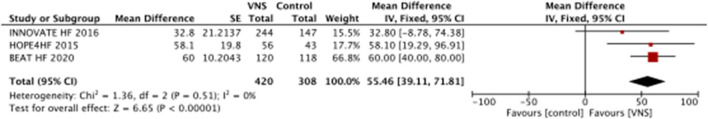
Improvement of the 6-min walking test during follow-up between VNS and control groups for management of chronic heart failure with reduced ejection fraction. VNS, vagus nerve stimulation.

Six trials measured this parameter. In the three RCTs, there was a significant increase in the distance achieved after VNS compared to control (MD, 55.46 m; 95% CI, 39.11–71.81; *p* < 0.0001) (Figure 5). In the other three prospective studies, there was also a significant improvement in the 6-min WT distance, ranging from 41 to 60 m.

#### NT-proBNP

**Figure 6:**
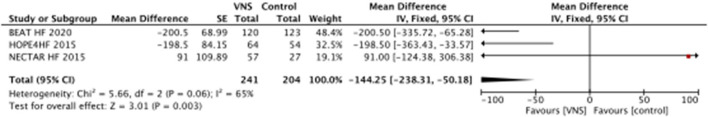
Improvement of NT-proBNP levels during follow-up between VNS and control groups for management of chronic heart failure with reduced ejection fraction. VNS, vagus nerve stimulation.

NT-proBNP levels were evaluated in three RCTs. There was an important decrease in levels in VNS groups when compared to controls (MD, −144.25; 95% CI, −238.31 to −50.18; *p* = 0.003) (Figure 6).

#### Adverse Events Related to the Procedure

Only one prospective study reported 10 AERP, including three deaths. In the RCTs, a total of 130 AERP occurred, with 70 deaths (6%). Therefore, freedom from AERP was seen in 89.3% of patients implanted with the VNS device.

## Discussion

This systematic review and meta-analysis showed an overall beneficial effect of the use of invasive VNS in patients with chronic heart failure and reduced ejection fraction. Altogether, the seven clinical trials included demonstrated a significant improvement in the functional NYHA class, QoL, 6-min WT, and NT-proBNP levels, with some adverse effects but no impact on mortality.

No differences in mortality were noted in any of the studies analyzed. This was not surprising as none of the studies were designed to detect differences in mortality, which would require a much larger number of patients since death rates with optimal medical therapy are around 3.8% according to a large clinical trial (22). The annual mortality rate is also higher in symptomatic patients, and some predictors of poor prognosis and increased mortality include systolic blood pressure <115 mmHg, serum creatinine >2.7 mg/dL, serum urea over 15 mmol/L, NT-pro-BNP exceeding 986 pg/ml, and LVEF under 45% (23). All patients involved in this meta-analysis presented LVEF <40% and NYHA class II or III, but they were also in full medical therapy, and differences in mortality in this setting were not expected, considering the small sample sizes.

A few cardiac parameters were assessed by the three RCTs. No significant improvement (two RCTs) or a trend to positive results (11) was observed in LVESV. This was similar to the results of an animal study where VNS effectively improved left ventricular function and remodeling (24). The three prospective studies analyzed corroborated this finding (4, 18, 19), with significant differences noted in LVESV before and after VNS. No differences were observed in LVEDV before and after VNS in any of the included studies. In two prospective studies, VNS leads to a decreased HR at 6 and 12 months after the procedure (4, 18). The NECTAR HF (21) study analyzed heart rate variability to assess the autonomic status (25) and found no differences before and after VNS, but patients were on beta blockers, which could have affected the measurements. Two prospective studies (4, 19) showed significant improvement on LVEF after VNS, but this was not analyzed by the RCTs. LVEF could fluctuate in repeated measurements or recover after treatment (26), blunting the borders between proposed categories of HF and should not be used as a surrogate marker of left ventricular systolic function.

New York Heart Association functional class and QoL improved after VNS in all studies (4, 14, 18–22). These positive effects demonstrate that most patients became less symptomatic and more capable of day-to-day activities after VNS treatment. A 6-min walking test was performed in six of the seven studies analyzed with a significant increase in walking distance in patients treated by VNS (4, 14, 15, 18–20). These findings align with the improvement in NYHA class and QoL observed, pointing that those patients became physically fitter after vagal stimulation.

An important finding of this meta-analysis was the significant decrease in NT-proBNP levels in patients with HFrEF treated with VNS, given the correlation between this biomarker and clinical outcomes in patients with heart failure. Indeed, NT-proBNP levels independently predict event-free survival in patients with systolic heart failure (27). Also, the NT-proBNP level is an objective measure, reinforcing the beneficial effects of VNS.

Adverse events definitions were different between studies. In the RCTs, they were defined as: (1) death and/or (2) hospitalization due to worsening heart failure, whereas, in the prospective studies, they were defined as any serious adverse event. Thus, the CARDIOFIT study (4) presented a high number of adverse events, but most of them did not meet the criteria used by the other studies, and 19 of the 26 events reported were not related to the procedure. In this systematic review and metanalysis, the overall rate of adverse effects related to the procedure was 10.7%. Importantly, mortality rates were similar between VNS and control. Some of the adverse events were related to the implantation of the vagal device, which has been previously described (3, 28).

Transcutaneous auricular VNS (ta-VNS) is a viable and non-invasive alternative with fewer side effects than invasive electrode implantation (29). A previous study identified that low-level transcutaneous electrical stimulation of the auricular branch of the vagus nerve was an effective modality for non-invasive autonomic neuromodulation in the beagle dog post-myocardial infarction mode (30), and it could activate the afferent and efferent vagal nerve and modulate intrinsic cardiac autonomic nervous system to achieve cardioprotective effect (31). Moreover, low-level transcutaneous electrical stimulation of the auricular branch of vagus nerve treatment was tolerated and convenient for ambulatory patients and, especially, for patients who could not have pharmacological therapies. It was feasible that non-invasive VNS may be useful for a large population of patients with HF (32). We are conducting a trial, registered in Brazilian Registry of Clinical Trials (ReBEC) under the number RBR-77wqymk (https://ensaiosclinicos.gov.br/rg/RBR-77wqymk) to investigate the effects of ta-VNS on patients with heart failure by comparing heart rate variability, NYHA functional class, 6-min walk test, and quality of life before and after 4 weeks of ta-VNS, five times a week.

The use of ta-VNS has been investigated in patients with paroxysmal atrial fibrillation (33, 34) without HF with promising results. The use of ta-VNS suppressed the arrhythmia and significantly decreased systemic levels of pro-inflammatory cytokines (tumor necrosis factor alpha and C-reactive protein). Therefore, future studies should evaluate ta-VNS as a mode for vagal stimulation in patients with chronic heart failure and reduced ejection fraction (HFrEF).

There were some limitations in the present systematic review and meta-analysis: (1) small number of studies included, which demonstrate the paucity of RCTs to evaluate the effects of vagal stimulation in this specific population, (2) heterogeneity in the objectives or primary outcomes of each study, and (3) no evidence regarding the etiology of the HF in most of the studies, something we know that may elicit different prognosis.

## Conclusions

In patients with chronic heart failure and reduced ejection fraction, the use of invasive vagal stimulation was associated with improvement of NYHA functional class, quality of life, 6-min walking test distance, and NT-proBNP levels, with a high grade of evidence. VNS was associated with some adverse events but had no impact on mortality. However, these results are limited to a small number of studies using variable outcomes. Thus, larger investigations using standardized methods and important outcomes are required. Also, non-invasive transcutaneous auricular vagal nerve stimulation (ta-VNS) is a viable alternative that may improve outcomes with less adverse events but needs further investigation.

## Data Availability Statement

The raw data supporting the conclusions of this article will be made available by the authors, without undue reservation.

## Author Contributions

LS and FS designed the study, conducted the research, and wrote the article. SC, EM, and GS contributed to the revision of the article for important content. LS, FS, PC, MS, and EF prepared the figures and helped to analyze and interpret the data. All authors have read and approved the final version of the article.

## Conflict of Interest

The authors declare that the research was conducted in the absence of any commercial or financial relationships that could be construed as a potential conflict of interest.

## Publisher's Note

All claims expressed in this article are solely those of the authors and do not necessarily represent those of their affiliated organizations, or those of the publisher, the editors and the reviewers. Any product that may be evaluated in this article, or claim that may be made by its manufacturer, is not guaranteed or endorsed by the publisher.
